# Long-Range Hydrogen Gas Measurement via Raman and Rayleigh–Brillouin Backscattering

**DOI:** 10.3390/s26175620

**Published:** 2026-09-04

**Authors:** Byoungjik Park, Jaeung Sim, Won Bo Cho, Hwi Seong Kim, In Ju Hwang

**Affiliations:** 1 Department of Fire Safety Research, Korea Institute of Civil Engineering and Building Technology (KICT), 182 Beon-Gil, Mado-Ro, Mado-Myeon, Hwaseong-si 18544, Gyeonggi-do, Republic of Korea; 2 Vimtech Co., Ltd., 201, Songpa-daero, Songpa-gu, Seoul 05854, Republic of Korea

**Keywords:** hydrogen gas, remote detection, real time measurement, raman scattering, brillouin scattering

## Abstract

**Highlights:**

- UV Raman lidar module designed for non-contact stand-off hydrogen gas detection.
- Brillouin scattering channel extends detection range to 30 m.
- Simultaneous Raman and Rayleigh–Brillouin acquisition using 360 nm UV source.
- PLS regression yields *R*^2^ = 0.97–0.98 at distances from 1 to 30 m.
- Hydrogen detected at 10–1000 ppm with SEC of 40–70 ppm across all distances.

**Abstract:**

Hydrogen (H^2^) is a promising energy carrier, but its wide flammability range requires rapid and reliable leak detection. In this study, we developed a non-contact stand-off ultraviolet (UV) light detection and ranging (lidar) system that simultaneously acquires Raman and Rayleigh–Brillouin backscattering signals for long-range H^2^ measurement. A 360 nm UV excitation source was used, and the system was evaluated at distances of 1, 3, 5, 10, 20, and 30 m using standard gases containing 10–1000 ppm H^2^. Quantitative analysis based on partial least squares (PLS) regression showed high linearity across the full distance range, with coefficients of determination (*R*^2^) of 0.97–0.98 and standard errors of calibration (SECs) of 40–70 ppm. The Rayleigh–Brillouin channel provided a useful complementary signal, particularly at longer distances, improving the robustness of concentration prediction when combined with the Raman response. These results demonstrate the feasibility of real-time, long-range H^2^ monitoring in large spaces and support the use of combined Raman and Rayleigh–Brillouin backscattering for safety monitoring in hydrogen-related facilities.

## 1. Introduction

Hydrogen gas is a highly promising alternative energy source, with growing applications in transportation and power generation [1,2,3]. However, its wide flammability range (4–75% in air) makes rapid and accurate leak detection essential for safety [4,5,6]. The development of reliable hydrogen sensors is therefore critical in any environment where hydrogen is produced, stored, or utilized [7,8,9].

Contact-based sensors have been widely employed for hydrogen detection using various transduction mechanisms [4,10,11,12]. While suitable for confined indoor spaces, contact sensors are inadequate in large open environments where hydrogen disperses rapidly at low concentrations [13,14,15]. Non-contact optical methods using stand-off laser sources offer a practical solution for large-area monitoring [16,17,18,19]. In particular, Raman scattering spectroscopy has been employed for several decades for molecular-level gas analysis [20,21], Rayleigh–Brillouin scattering has been investigated as a complementary molecular scattering channel [22,23,24,25], and combined approaches exploiting both mechanisms have also been reported [26]. Spectral separation of Rayleigh–Brillouin and Mie scattering components is fundamental to extracting molecular gas signatures from lidar backscatter [27,28].

Most hydrogen Raman lidar systems have operated in the 532 nm band, which suffers from strong ambient-light interference. Ultraviolet excitation (300–400 nm) significantly reduces this interference and improves the signal-to-background ratio [29,30]. More recently, excitation at 442 nm has been proposed as a compromise between the visible and UV bands; such intermediate-wavelength systems benefit from higher detector responsivity and lower source cost, but remain more susceptible to ambient light and gain less from the λ−4 scaling of the scattering cross-section than the 360 nm excitation adopted here. Nevertheless, the Stokes–Raman signal decreases rapidly with distance, limiting most studies to measurement ranges below 10 m. To extend detection to 30 m, we introduced an additional collection lens and exploited the Brillouin scattering zone—located spectrally between the Rayleigh peak and the Raman bands—as a supplementary detection channel [31]. An earlier study using a 425 nm filter to capture vibrational Raman scattering at 4152 cm^−1^ demonstrated that signal intensity saturates beyond a certain range [32]; the present work addresses this by simultaneously acquiring the Rayleigh–Brillouin band and the rotational Raman band spanning 395–415 nm for a 360 nm UV source.

This study investigated simultaneous Raman and Rayleigh–Brillouin scattering for stand-off hydrogen gas detection at distances of 1–30 m and concentrations of 10–1000 ppm. Quantitative analysis was performed using partial least squares (PLS) regression with Unscrambler 7.5 (CAMO, USA). The results demonstrate the feasibility of real-time, long-range hydrogen detection, with potential applications in hydrogen energy infrastructure and large-space safety monitoring [33].

## 2. Methodology

### 2.1. Experimental Setup

**Figure 1:**
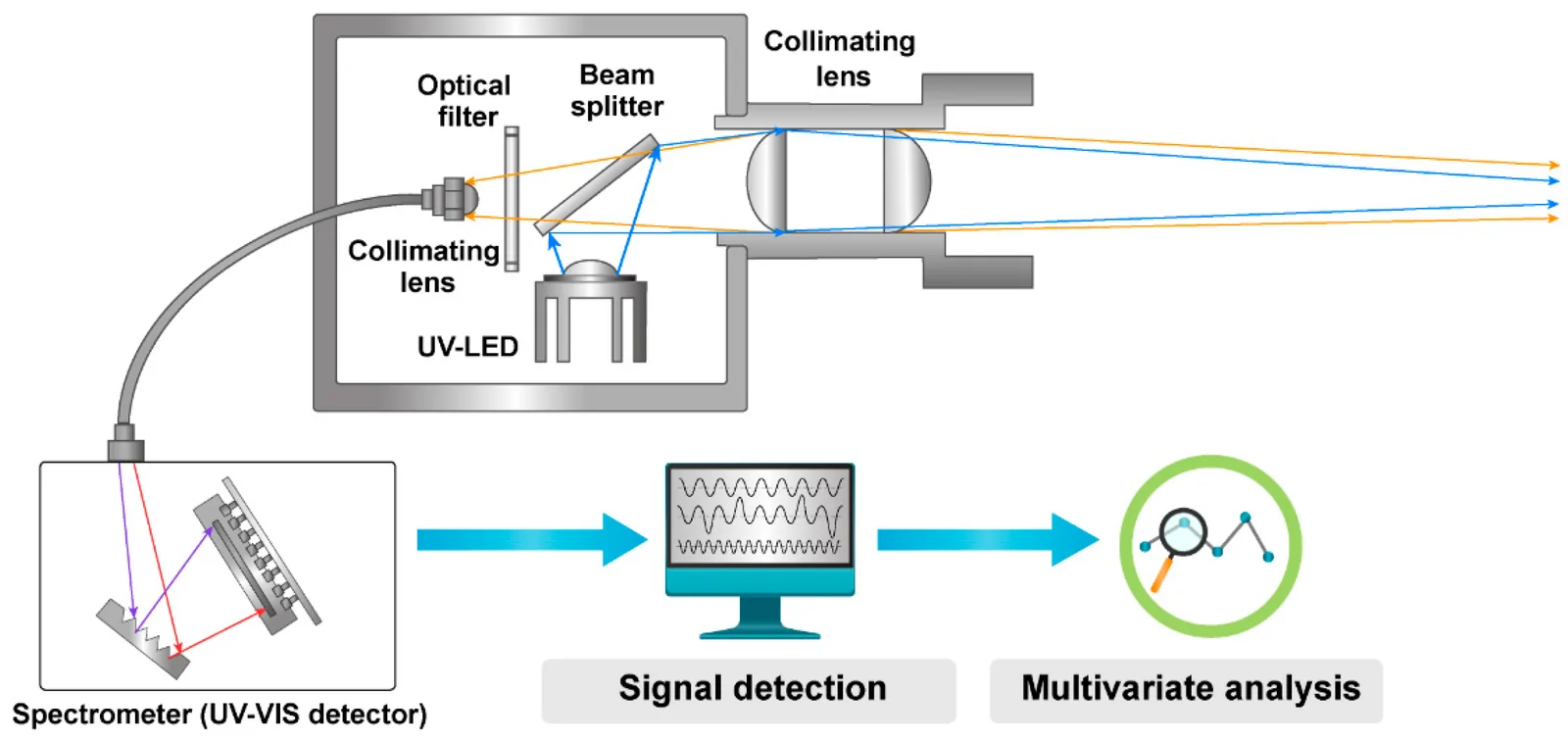
Raman lidar hydrogen measurement module. Schematic diagram of the non-contact hydrogen measurement system configured for long-range lidar operation in large spaces, showing the 360 nm UV laser source, collection optics, spectral filter, and detector assembly.

The proposed measurement device, designed to measure hydrogen using long-range lidar in a large space, was configured as shown in Figure 1. A 360 nm (wavelength FWHM: 50 nm, output power: 10 W; COB 10W LED, Guhon, China) UV light-emitting diode (LED) source was used to excite both Rayleigh–Brillouin and Raman scattering. The UV source beam was directed toward the measurement volume by a beamsplitter (reflection:transmission ratio 50:50, UV-fused silica, 25.4 mm diameter, BSW 20, Thorlabs, USA), and the backscattered light returning along the same axis was transmitted through the beamsplitter to the collection optics. To detect the backscattered signal as the light from the light source collided with the hydrogen gas, a plano-convex lens (UV-fused silica, 25.4 mm diameter, Thorlabs, USA) was used for collimation, allowing the scattered signal to be received effectively, and beam was focused by two plano-convex lenses. As a result, remote measured function was possible. Further, to detect the characteristic signal, the Raman signal collected by the collimating lens was passed through a 400 nm optical filter and an optical fiber (core diameter: 1000 μm, material: quartz), which was measured using a spectrometer (UV–VIS range, USB 4000, Ocean Optics, USA). Because the spectrometer used here was capable of measuring from 200 to 1100 nm, a grating (1200 grooves/mm, concave grating) and a multi-array silicon detector (1024 CCD detector) were used for real-time measurements (Figure 1). Each spectrum was acquired with an integration time of 1000 ms and 5 accumulated scans per measurement, and the spectral resolution of the spectrometer was 1.5 nm.

The key optical parameters of the system are summarized in Table 1.

### 2.2. Experimental Procedure

**Figure 2:**
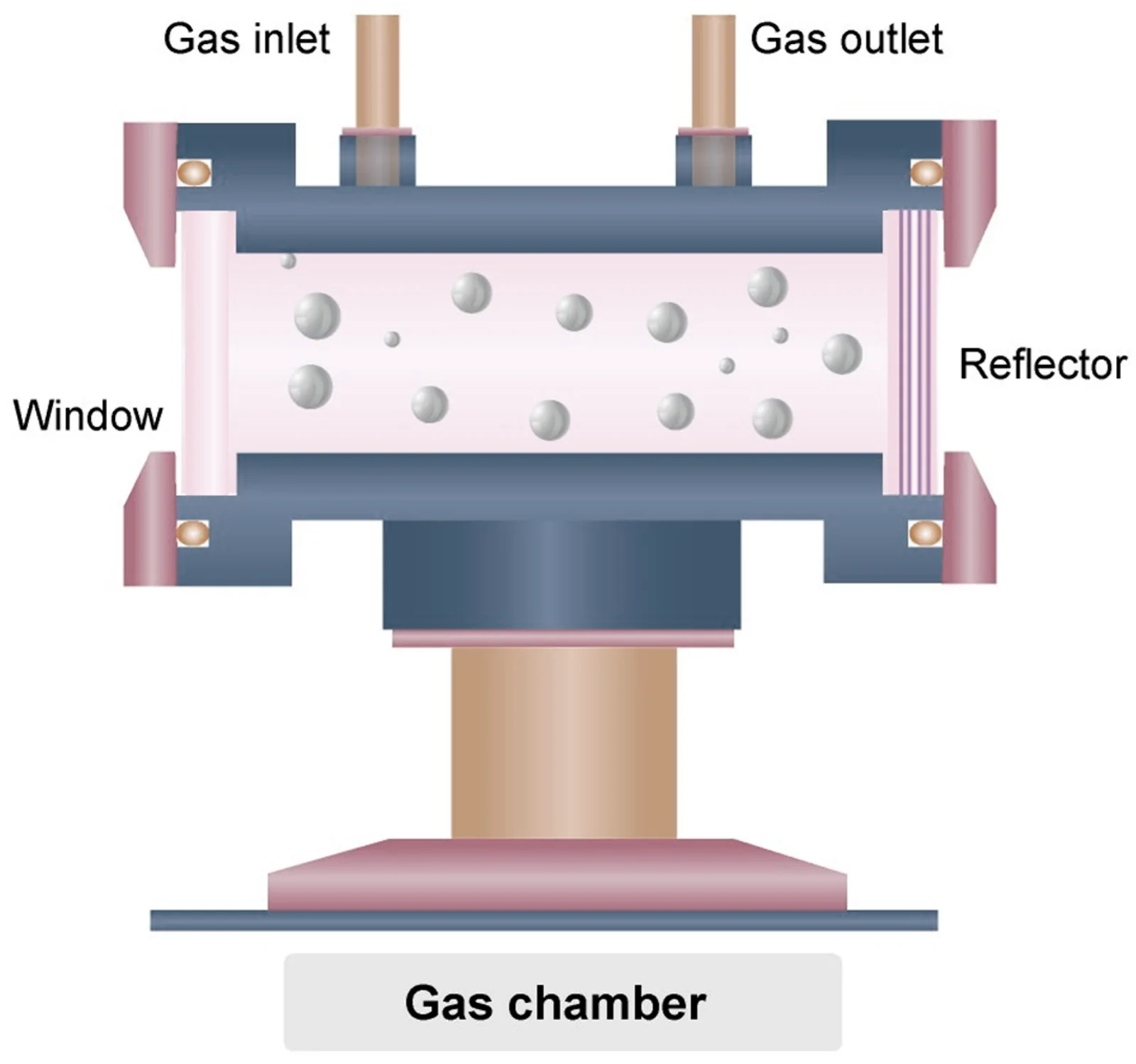
Gas measurement chamber. Experimental chamber used for controlled introduction of hydrogen standard gas, with optical access ports positioned at the far end to enable stand-off backscattering measurements.

For the qualitative and quantitative analyses of hydrogen gas, a standard gas with hydrogen in a nitrogen balance at defined concentrations was used. To measure the concentration of hydrogen gas under conditions where it was maintained at a constant level in the atmosphere, a chamber made of stainless steel [23] with a diameter of 100 mm and a length of 210 mm was used, which was filled with hydrogen standard gas according to the concentration for measurement. A Raman light source was designed such that its light passed through the chamber and then was sent to the detector via a window using backscattering. Because the light source and detector used the reflection method, a quartz window was installed on one side, and a stainless-steel plate with a thickness of 100 mm was manufactured and installed on the other side (Figure 2). Specular and stray reflections within the illumination path were suppressed by the off-axis collection geometry and by the stainless-steel end plate of the chamber, and residual elastic stray light was further rejected by the 400 ± 15 nm bandpass filter, so that the detected spectra were not dominated by direct illumination.

The experiment utilized standard gases (Rigas, Korea) with hydrogen gas concentrations of 10, 50, 100, 500, and 1000 ppm within a nitrogen environment. With all valves of the chamber maintained open, standard gas was introduced into the chamber via the inlet, while gas was permitted to escape continually through the outlet to mitigate fluctuations in gas concentration caused by pressure differences. The experiment was conducted following the gas stabilization concentration during the period of gas injection into the chamber.

### 2.3. Data Processing

Prior to analysis, a background spectrum was acquired at each measurement distance with the chamber filled with pure nitrogen, using the same acquisition parameters as for the sample measurements, and was subtracted from every sample spectrum (Spectrasuite software, Oceanoptic, USA). The background-corrected spectra were then smoothed and differentiated using the Norris derivative method, and the first-derivative spectra were used to verify the representative wavelengths (Section 3.1).

Quantitative calibration was performed by partial least squares (PLS) regression using Unscrambler 7.5 (CAMO, USA). The spectral intensities in the Rayleigh–Brillouin and Raman zones formed the X-block, and the standard-gas concentrations (10–1000 ppm) formed the Y-block; the models were evaluated by cross-validation, and their performance was expressed by the coefficient of determination (*R*^2^), the standard error of calibration (SEC), and the root-mean-square error of calibration (RMSEC), as defined in Section 3.3.

## 3. Results and Discussion

### 3.1. Measurement of Hydrogen Spectrum by Rayleigh–Brillouin Scattering and Stokes–Raman Scattering of Rotational Energy

**Figure 3:**
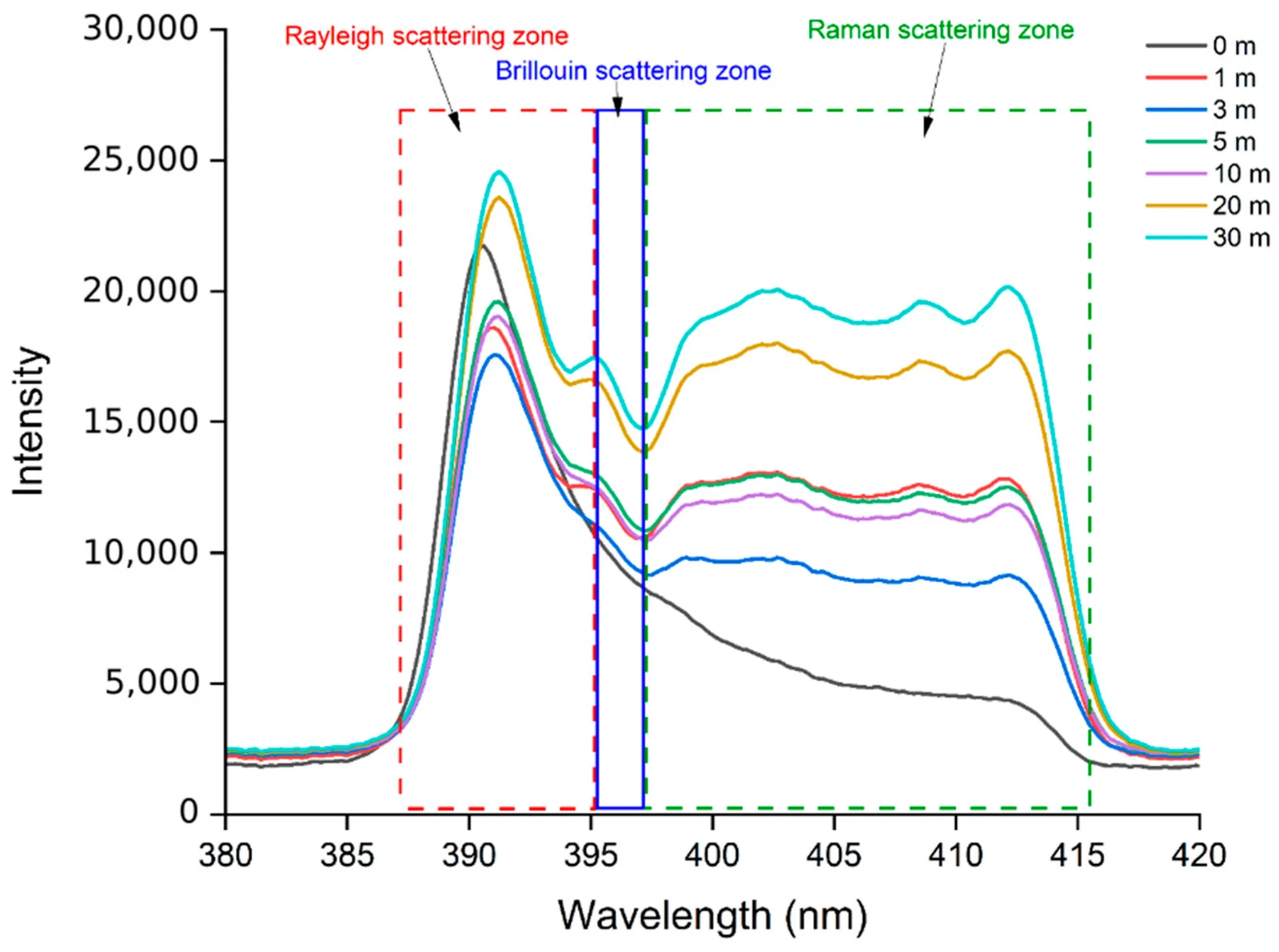
Measurement ranges of Brillouin scattering and Raman scattering (100 ppm hydrogen standard gas). Spectral intensity distribution across the Rayleigh–Brillouin (395–399 nm) and Raman (399–415 nm) zones as a function of measurement distance (0–30 m).

Backscattering occurs in three types of scattering, including Rayleigh scattering, which excludes the same wavelength range as that of the UV source and has the highest intensity among the scattered light. To minimize the wavelength range of the UV light source, Rayleigh-scattered light in the same wavelength range as that of the UV light source was cut off through a 400 nm (±15 nm) bandpass optical filter. The measured emission spectrum of the UV source, together with the transmission band of this filter confirming that direct source light within the 385–415 nm analysis window, is strongly suppressed. The scattered light then appears between the cutoffs of the Rayleigh scattering and Raman scattering zone, and this region is called the Brillouin scattering zone. Here, the Brillouin scattering wavelength range corresponds to a tiny area, from 0.1 to 6 cm^−1^, which was calculated to be in the 395 nm range. The region that included the Brillouin scattering and the cutoff of Rayleigh scattering was referred to as the Rayleigh–Brillouin scattering zone, and the wavelength range from 399 nm to 415 nm was classified as the broader Raman scattering zone (Figure 3). The representative wavelengths used throughout this study and their scattering-zone assignments are summarized in Table 2.

Figure 3 shows that the signal value is at its lowest at a distance of 0 m. From a distance of 1 m, the scattering intensities of both the Rayleigh–Brillouin scattering and Raman scattering zones increased. The signal value did not increase linearly with the distance. This non-monotonic behavior is attributed mainly to the geometric overlap function of the transmitter–receiver arrangement: at short range, the field of view of the collection optics does not fully contain the illuminated volume, so the collected signal first increases with distance as the overlap improves, and only at longer range do radiometric losses begin to dominate. Because the background level also rises with distance, all quantitative analyses were performed on background-subtracted spectra (Section 2.3). In the Rayleigh–Brillouin scattering and Raman scattering zones, Rayleigh scattering was cut off; however, the Rayleigh zone exhibited the highest intensity scattering among the three scattering zones. For wavelengths in the 391 nm range, the intensity at a distance of 3 m was 17,544 counts, whereas that at a distance of 30 m was the highest observed at 24,575 counts.

**Figure 4:**
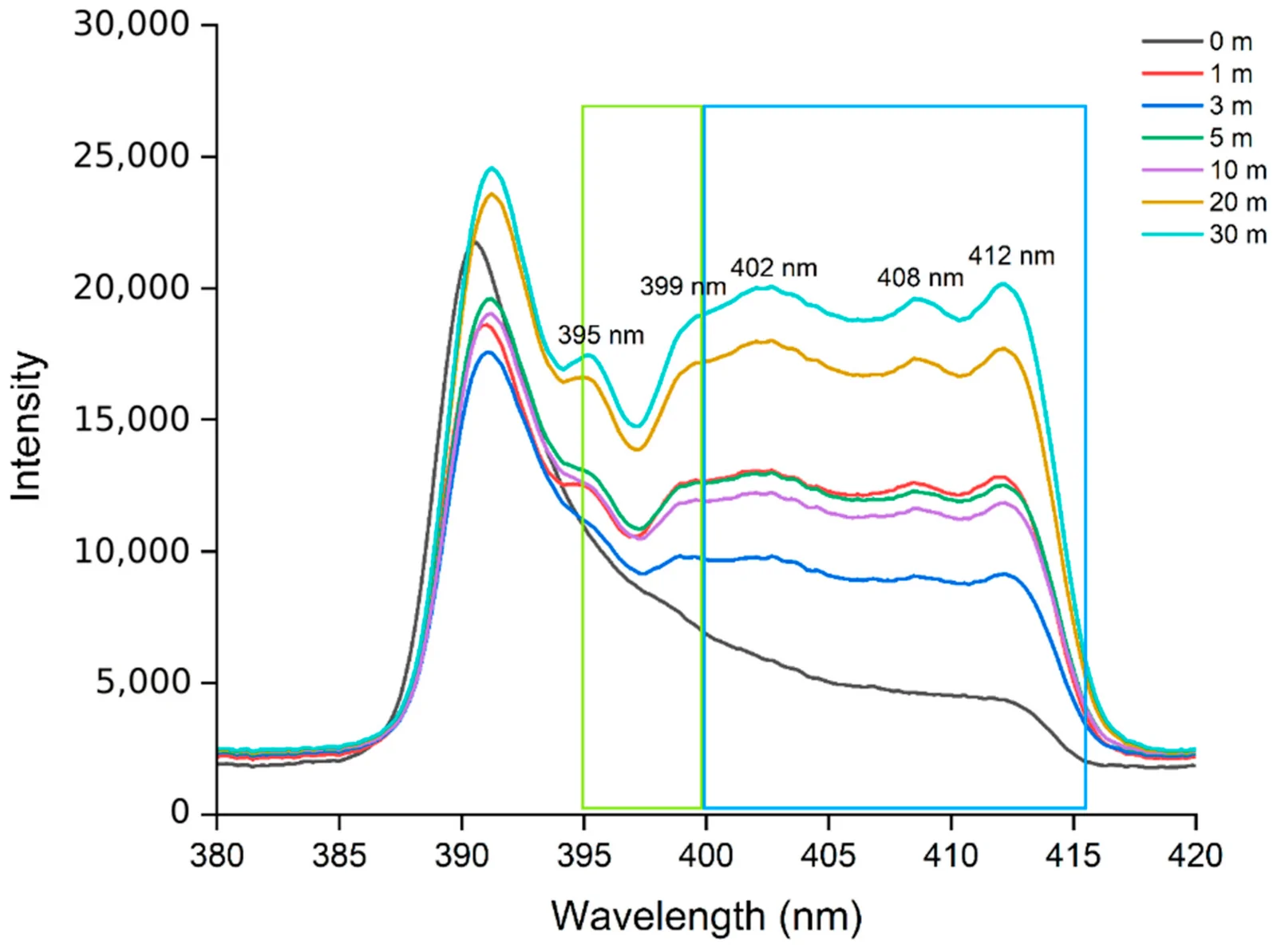
Representative wavelengths for Rayleigh–Brillouin scattering and Raman scattering. Characteristic wavelengths identified in each scattering zone (391, 395, 397 nm for Rayleigh–Brillouin; 402, 408, 412 nm for Raman) plotted as a function of distance.

Meanwhile, the wavelength range for Raman scattering with rotational energy for hydrogen in the Raman scattering zone was 402 nm, 408 nm, and 412 nm (corresponding to rotational Raman shifts of 357, 592, and 819 cm^−1^). In this experiment, these wavelengths were identified as representative wavelengths. The hydrogen Raman intensities in the Rayleigh scattering zone at 391 nm and in the Brillouin scattering zone at 395 nm and 397 nm were linked in the same way (Figure 4). Because these Raman lines originate from rotational transitions of hydrogen, their spectral positions are fixed by the molecular rotational constants and are insensitive to concentration or measurement distance under the present conditions; only their intensities vary with the number density of hydrogen molecules.

**Figure 5:**
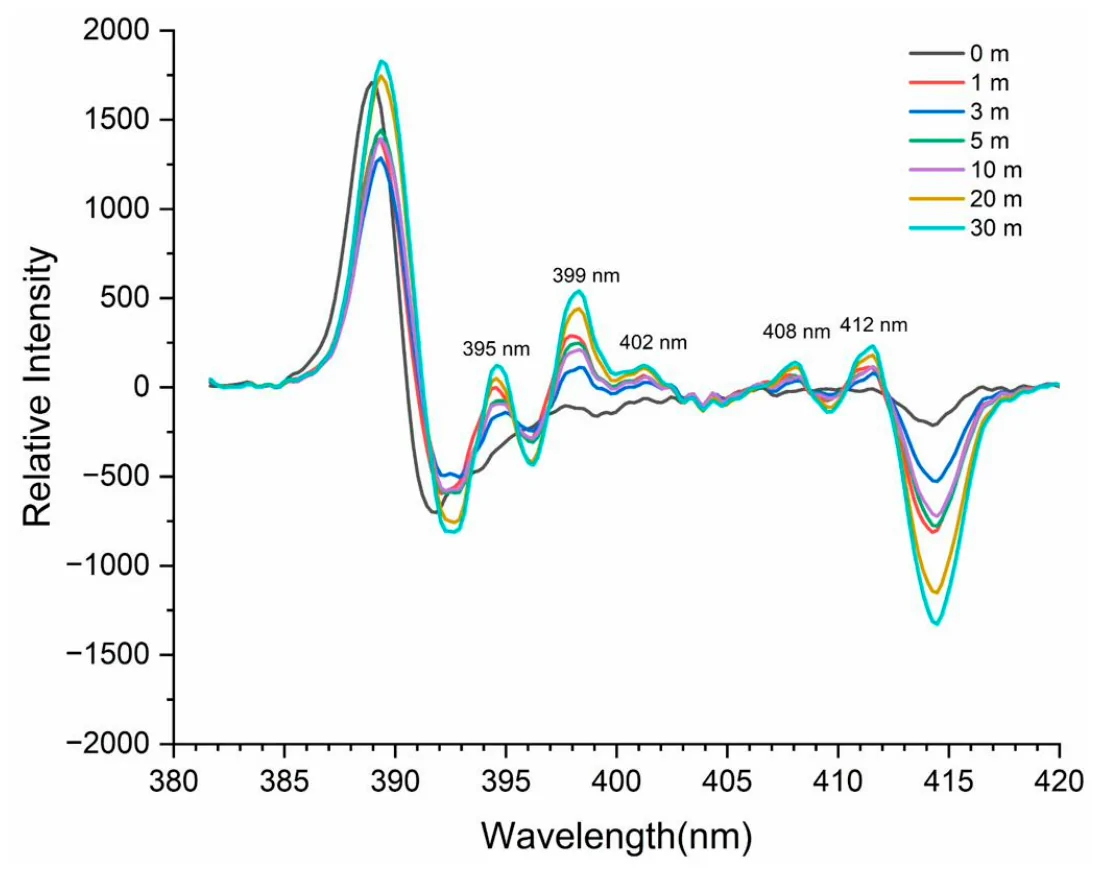
Verification of wavelength signal through the first derivative of the scattering signal. Verification of wavelength signal through the first derivative of the scattered signal. First-derivative spectra used to confirm representative scattering wavelengths and to discriminate hydrogen signal features from background.

The first derivative of the scattered signal was used to determine the normalcy of the signals across five wavelength bands. The first derivative was calculated using the Norris method and the results showed that 395 nm, 399 nm, 402 nm, 408 nm, and 412 nm were all wavelength signals (Figure 5).

The wavelength range of Rayleigh–Brillouin scattering at 390 nm is the region where the intensity increases sharply, which was confirmed to be a strong signal based on the first derivative. In the vicinity of the Raman scattering zone at 415 nm, the signal shows a rapid decrease in intensity, appearing as a negative value in the first derivative.

### 3.2. Measurement of Hydrogen Raman Spectrum Changes Using Standard Gases by Concentration

After the hydrogen standard gas (concentration = 10, 50, 100, 500, or 1000 ppm) was placed inside the fabricated gas chamber, the spectral changes were observed using Rayleigh–Brillouin scattering and Raman scattering measurement sensors. The distance between the measurement sensors and the chamber was varied from 1 m to 30 m, and the characteristics of the changes in the Rayleigh–Brillouin and Raman scattering spectrum with respect to concentration were examined at representative distances of 1, 5, and 30 m.

#### 3.2.1. Measurement of Rayleigh–Brillouin and Raman Spectrum Changes According to Hydrogen Concentration at a Measurement Distance of 1 m

**Figure 6:**
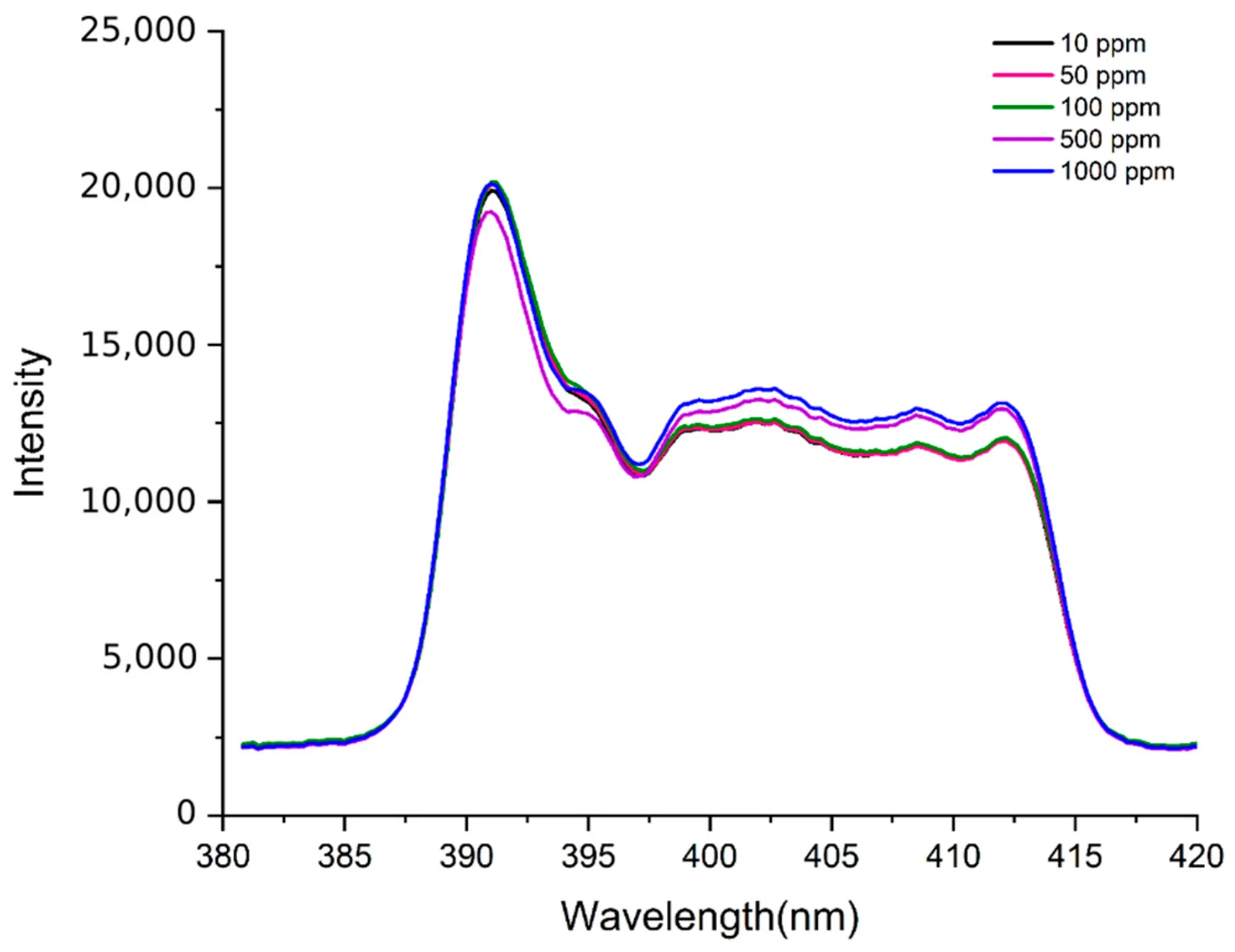
Rayleigh–Brillouin and Raman spectra according to hydrogen concentration at a distance of 1 m. Rayleigh–Brillouin and Raman spectra according to hydrogen concentration. Spectral intensities in (**top**) the Rayleigh–Brillouin zone and (**bottom**) the Raman zone, plotted as a function of hydrogen standard gas concentration (10–1000 ppm) at selected measurement distances.

In this part of the experiment, the measurement distance between the chamber containing the hydrogen standard gas and the Rayleigh–Brillouin and Raman hydrogen sensors was fixed at 1 m. The resulting measurements, with respect to the concentration of the standard gas, are shown in Figure 6.

Overall, the spectrum with the highest intensity was the Rayleigh scattering zone, with an intensity of 20,000 counts. In contrast, the background spectrum intensity was 2297 counts at 383 nm. The intensity for each wavelength was 13,700 counts at 395 nm in the Rayleigh scattering zone and 12,990 counts at 399 nm in the Brillouin scattering zone. The rotational energy of the Raman scattering zone was measured to be 13,430 counts at 402 nm, 12,820 counts at 407 nm, and 13,010 counts at 411 nm. At low hydrogen gas concentrations (10 ppm and 50 ppm) the spectral intensity was lower than that at 100 ppm, whereas at higher concentrations (500 ppm and 1000 ppm), the spectral intensity was higher than that at 100 ppm.

#### 3.2.2. Measurement of Rayleigh–Brillouin and Raman Spectrum Changes According to Hydrogen Concentration at a Measurement Distance of 5 m

**Figure 7:**
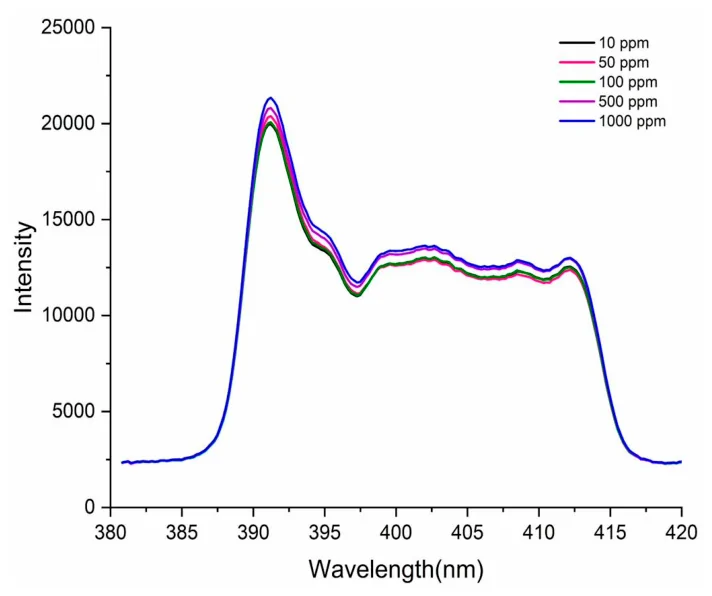
Rayleigh–Brillouin and Raman spectra according to hydrogen concentration at a distance of 5 m. Rayleigh–Brillouin and Raman spectra according to hydrogen concentration and distance (overall spectrum). Full-range backscatter spectra illustrating the combined Rayleigh–Brillouin and Raman signals for varying hydrogen concentrations across measurement distances of 1–30 m.

The Rayleigh–Brillouin and Raman spectra were measured at a distance of 5 m between the measurement sensor and gas chamber, using the same measurement method and system as in the previous experiment. In the overall spectrum shown in Figure 7, the background spectrum intensity was 2493 counts, which was higher than that at a measurement distance of 1 m. The highest Rayleigh scattering intensity was 21,350 counts, which was higher than that of the scattering zone for a measurement distance of 1 m. The measured intensities at each wavelength were 14,190, 13,230, 13,580, 12,890, and 13,010 at 395 nm, 399 nm, 402 nm, 407 nm, and 411 nm, respectively. In the Rayleigh–Brillouin scattering zone, the intensity of the scattering zone was higher than that at a measurement distance of 1 m, whereas the Raman scattering zone exhibited equal or higher intensity levels.

#### 3.2.3. Measurement of Rayleigh–Brillouin and Raman Spectrum Changes According to Hydrogen Concentration at a Measurement Distance of 30 m

**Figure 8:**
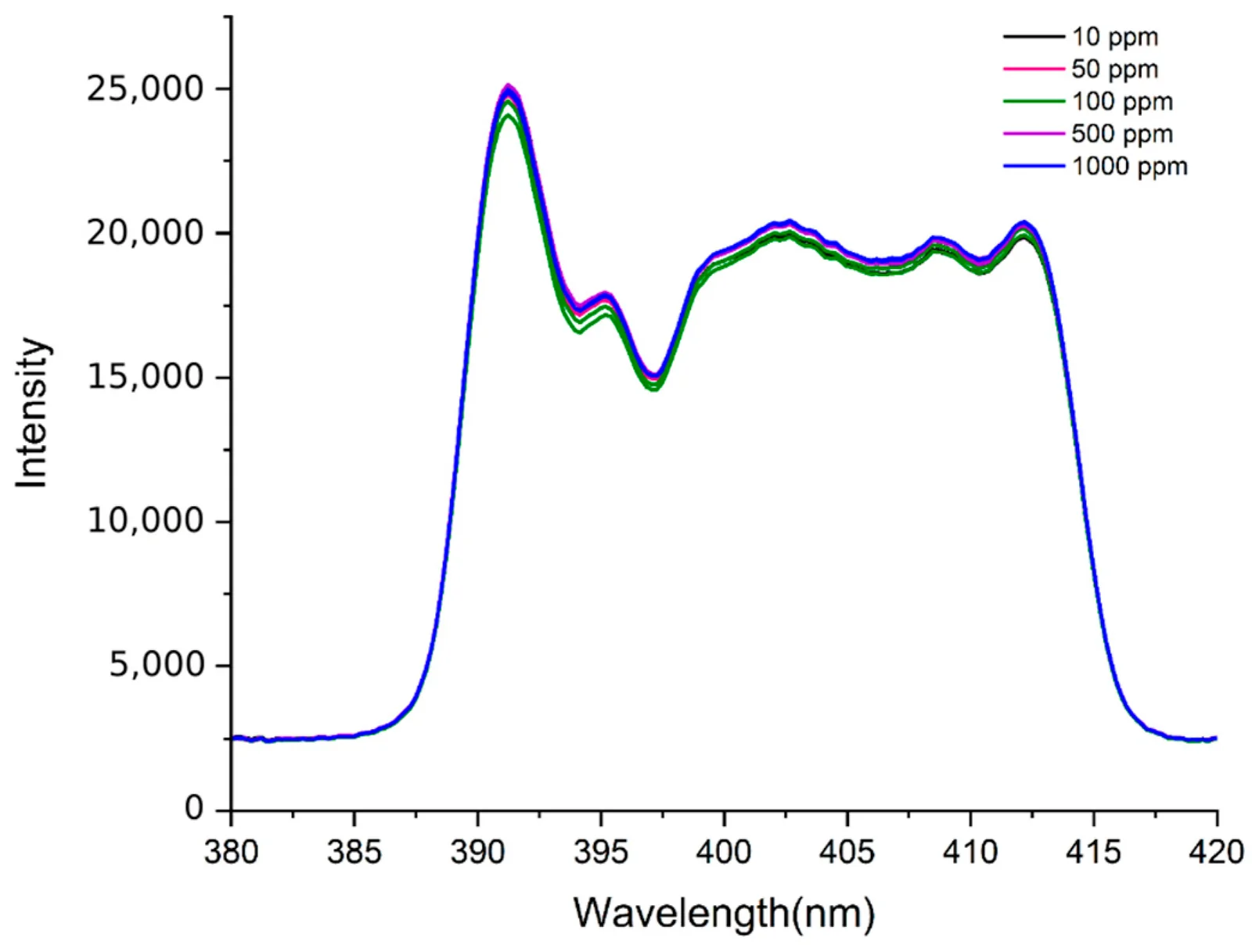
Rayleigh–Brillouin and Raman spectra according to hydrogen concentration at a distance of 30 m. Rayleigh–Brillouin and Raman spectra according to hydrogen concentration (background-subtracted). Background-corrected scattering spectra in both the Brillouin and Raman zones, confirming the proportional relationship between signal intensity and hydrogen concentration.

The hydrogen gas was measured at a distance of 30 m between the measurement sensor and gas chamber using the same measurement method and system as in the previous experiment. The reason for experimenting with such long-distance measurements is that the intensity of the measurement signal changes with distance; thus, a measurable distance must be determined. Based on the measurement results shown in Figure 8, the background spectrum intensity increases to 2683 counts. Furthermore, the highest intensity for the Rayleigh scattering zone was 24,970 counts, which was higher than that at measurement distances of 1 and 5 m. The intensities were 17,860, 19,260, 20,400, 19,850, and 20,432 counts at 395, 399, 402, 407, and 411 nm, respectively. These results indicate a higher intensity scattering zone in the longer-wavelength range than the results at 1 and 5 m. In other words, as the measurement distance increased, the measured scattering intensity increased, indicating the advantage of this wavelength range for long-distance measurements.

### 3.3. Quantitative Analysis of Hydrogen Raman Spectra Through Multivariate Analysis Methods

Multivariate analysis is a statistical technique that contrasts with univariate analysis in that it considers the relationships between multiple variables simultaneously [34]. One example of multivariate analysis is PLS, which derives latent variables through a repeated process of calculating loading values, considering the correlation with the dependent variable *Y* when deriving components that explain the variance in the *X* and *Y* space. PLS has high predictive power and excellent noise reduction effects [35]. Because both independent and dependent variables are considered, this technique exhibits greater stability than traditional multivariate analysis models that consider only the independent variables. The PLS model can be expressed in matrix form as follows:

*X* = *TP* + *Ex* (1)

**Figure 9:**
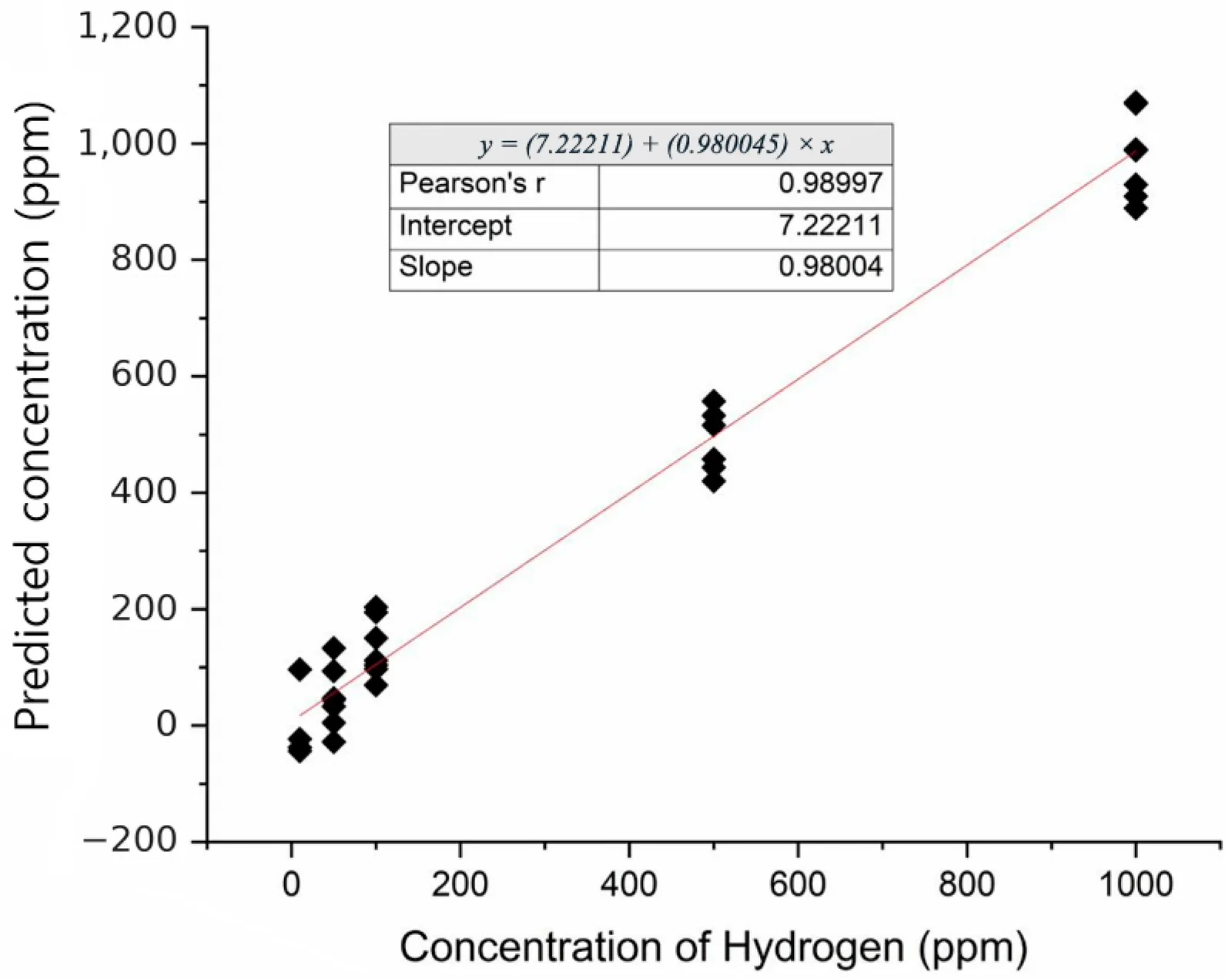
Verification of linearity in the regression analysis at a measurement distance of 1 m. PLS regression calibration plot showing the relationship between measured and predicted hydrogen concentrations (*R*^2^ = 0.98, calibration slope = 0.98, SEC = 55.36 ppm).

*Y* = *UQ* + *Ey* (2)

where *X* denotes the matrix of measured spectra and *Y* the matrix of reference hydrogen concentrations; *T* and *P* denote the scores and loadings of *X*, respectively; *U* and *Q* denote the scores and loadings of *Y*, respectively; and *Ex* and *Ey* denote the residual matrices. The score matrix *T* of the spectrum *X* and the score matrix *U* of the measured concentration *Y* have the following relationship:

(3) $$B={\left(T^{T}T\right)}^{-1}T^{T}U$$

where *B* denotes the regression coefficients of PLSR, and *T* denotes the transpose of the vector. With this least-squares method, the Brillouin–Raman scattering spectra and standard gases were quantitatively analyzed. Regression analysis by applying PLS to the results at a measurement distance of 1 m (Figure 9) showed high linearity (*R*^2^ = 0.98), with a slope of 0.98 for the calibration curve, and a standard error of calibration (SEC) of 55.36 ppm. The limit of detection was estimated as three times the standard deviation of the blank (nitrogen-filled chamber) divided by the calibration slope. The calibration points cluster into discrete groups along the concentration axis because the standard gases were prepared at 10, 50, 100, 500, and 1000 ppm, i.e., approximately logarithmically spaced levels.

After the hydrogen gas was quantified and analyzed at measurement distances of 3, 5, 10, 20, and 30 m, similar to that conducted for 1 m, the results were subjected to cross-validation. The statistical data for the linear equations, linear coefficients (*R*^2^), and SEC of each calibration curve are listed in Table 3.

The linear equation of the calibration curve is a first-degree equation, and the closer the linear coefficient is to 1, the higher the correlation, indicating its suitability for prediction.

(4) $$R^{2}={\left(\frac{\sum_{i=1}^{n}(y_{i}-\bar{y})({\hat{y}}_{i}-\bar{\hat{y}})}{\sum_{i=1}^{n}\sqrt{{\left(y_{i}-\bar{y}\right)}^{2}}\sum_{i=1}^{n}\sqrt{{\left({\hat{y}}_{i}-\bar{\hat{y}}\right)}^{2}}}\right)}^{2}$$

where *n* denotes the number of samples, *y_i_* the measured concentration of the *i*-th sample, ${\hat{y}}_{i}$ the concentration predicted by the model, and $\bar{y}$ the mean of the measured concentrations. To verify the accuracy of the predicted model, the root mean square difference between the calibration line and the predicted values was calculated as follows:

(5) $$RMSEC=\sqrt{\frac{{\sum_{i=1}^{N}\left({\hat{y}}_{i}-y_{i}\right)}^{2}}{\left(N-A-1\right)}}$$

where ${\hat{y}}_{i}$ is the value predicted by the calibration model, *N* is the number of calibration samples, and *A* is the number of PLS latent variables (factors) retained in the model. This result was obtained considering three aspects: the calibration equation, linear coefficient (*R*^2^), and SEC. For measurement distances of 1–5 m, the linear coefficient was 0.98, and for longer distances, the coefficient was 0.97, which was similar. Beyond the near field, the deviation (SEC) increases gradually with distance as the backscattered signal weakens; the slightly higher SEC at 1 m than at 3 and 5 m is attributed to near-field effects, namely incomplete geometric overlap and greater sensitivity to the detector dynamic range at close range (Section 3.1).

### 3.4. Loading Value via PLS for Hydrogen Standard Gas

**Figure 10:**
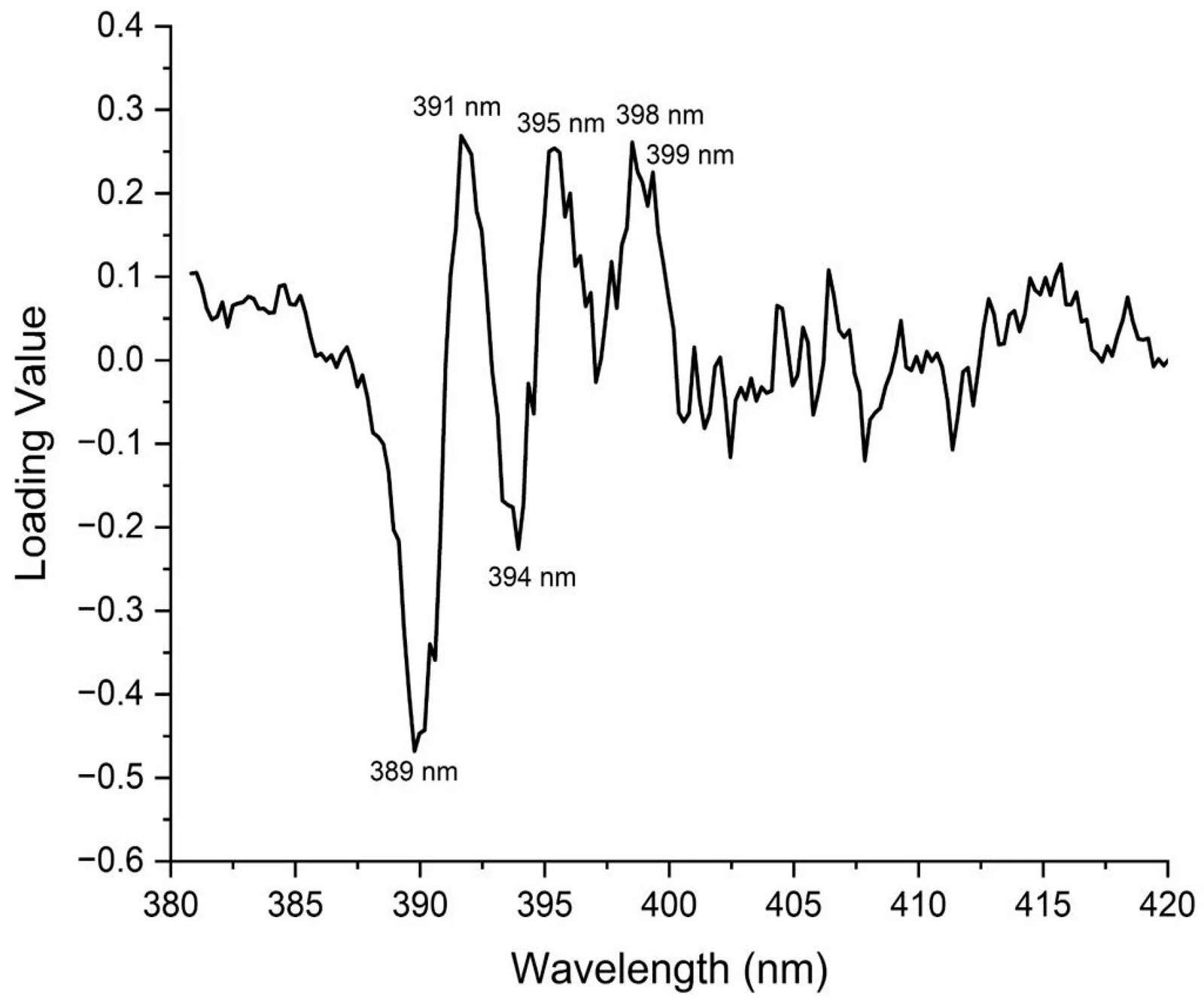
Loading values in calibration model for measurement distance of 1 m. PLS loading values as a function of wavelength, indicating the relative contribution of each spectral region (Rayleigh, Brillouin, and Raman zones) to the hydrogen concentration prediction model.

The high linearity observed in this study was further confirmed using PLS. The analysis enabled the determination of the scattering zone that had the greatest effect by examining the loading values obtained via PLS. The loading values for each wavelength, according to the hydrogen concentration, are shown in Figure 10.

Hydrogen gas concentration, whereas 391 nm and 395 nm were positively affected. Meanwhile, 398 nm and 399 nm, located at the boundary between the Rayleigh–Brillouin and Raman scattering zones, were also confirmed to have a positive effect, and the representative rotational Raman wavelengths identified in Section 3.1 (402, 408, and 412 nm) likewise showed positive loadings. These results suggest that all three regions affected the observed concentration of hydrogen. The loading value of the calibration line for a measurement distance of 1 m was the same as for the other measurement distances.

## 4. Conclusions

This study developed a non-contact Raman lidar hydrogen gas measurement module to measure hydrogen gas in large spaces. The potential applications of the developed sensor in hydrogen-related industries and gas measurements in large spaces were confirmed. The experimental results demonstrated that the developed sensor can accurately measure and detect hydrogen gas concentrations at various distances. The findings can be summarized as follows:

1. A 360 nm Raman light source was used for hydrogen gas measurements, and the changes in Raman scattering and Rayleigh–Brillouin scattering were observed.
2. Measurements were obtained at various distances using a 100 ppm hydrogen standard gas. In particular, as the distance increased from 0 m to 30 m, the scattering intensities of both the Rayleigh–Brillouin and Raman scattering zones increased. Notably, the Rayleigh scattering zone was correlated with distance.
3. The spectral results were verified based on the distance for each standard gas concentration, and the signal intensity was proportional to the gas concentration.
4. Quantitative analysis of hydrogen gas with respect to distance was performed based on the measured data. The results showed that the reliability was high for measurement distances from 1 m to 30 m, particularly based on the changes in the Rayleigh–Brillouin scattering zone along with the Raman scattering region.

This study proposed a method for long-distance analysis of hydrogen gas using a hydrogen measurement module that employs a Raman light source, utilizing both Raman scattering and Brillouin scattering to improve the reliability of extended measurements. Future research will focus on hydrogen measurement utilizing Raman sources to facilitate its application in the hydrogen industry.

## Figures and Tables

**Table 1:** Specifications of the main optical components.

Component	Specification
UV source	360 nm LED, FWHM 50 nm, output power 10 W
Beamsplitter	50:50 (R:T), UV-fused silica, Ø 25.4 mm
Collimating/focusing lenses	Plano-convex, UV-fused silica, Ø 25.4 mm, focal length 50 mm
Bandpass filter	400 ± 15 nm
Optical fiber	Quartz, core diameter 1000 μm, NA 0.22
Grating	Concave, 1200 grooves/mm
Detector	Si multi-array CCD, 1024 pixels
Spectrometer range/resolution	200–1100 nm/1.5 nm

**Table 2:** Representative wavelengths and their scattering-zone assignments.

Wavelength (nm)	Scattering Zone	Rotational Raman Shift (cm^−1^)
391	Rayleigh	–
395	Rayleigh–Brillouin	–
399	Rayleigh–Brillouin (boundary with Raman zone)	–
402	Raman	357
408	Raman	592
412	Raman	819

**Table 3:** Quantitative analysis by distance.

Measurement Distance (m)	Calibration Curve Equation	Linear Coefficient (*R*^2^)	SEC
1	Y = 0.98X + 7.2221	0.98	55.36
3	Y = 0.9868X + 4.7732	0.98	45.18
5	Y = 0.9842X + 5.3583	0.98	48.08
10	Y = 0.9755X + 8.7895	0.97	57.89
20	Y = 0.9747X + 8.5131	0.97	60.18
30	Y = 0.9691X + 10.976	0.97	60.26

Summary of PLS calibration results for each measurement distance (1–30 m), including the linear calibration equation, linear coefficient (*R*^2^), and standard error of calibration (SEC in ppm).

## Data Availability

The datasets supporting this study are not publicly available due to privacy reasons but can be made available from the corresponding author upon reasonable request.

## Abbreviations

H^2^	Hydrogen
KICT	Korea Institute of Civil Engineering and Building Technology
PLS	Partial least squares
PLSR	Partial least squares regression
SEC	Standard error of calibration
UV	Ultraviolet
UV-VIS	Ultraviolet–visible
